# Is Exon Skipping a Viable Therapeutic Approach for Vascular Ehlers–Danlos Syndrome with Mutations in *COL3A1* Exon 10 or 15?

**DOI:** 10.3390/ijms25168816

**Published:** 2024-08-13

**Authors:** Sasiwimon Utama, Jessica M. Cale, Chalermchai Mitrpant, Sue Fletcher, Steve D. Wilton, May T. Aung-Htut

**Affiliations:** 1 Department of Biochemistry, Faculty of Medicine Siriraj Hospital, Mahidol University, Bangkok 10700, Thailand; sasifern@hotmail.com (S.U.); chalermchai.mit@mahidol.edu (C.M.); 2 Centre of Molecular Medicine and Innovative Therapeutics, Murdoch University, Murdoch, WA 6150, Australia; jessica.cale@murdoch.edu.au (J.M.C.); s.fletcher@murdoch.edu.au (S.F.); s.wilton@murdoch.edu.au (S.D.W.); 3 Perron Institute for Neurological and Translational Science, The University of Western Australia, Nedlands, WA 6009, Australia; 4 Centre for Neuromuscular and Neurological Disorders, The University of Western Australia, Nedlands, WA 6009, Australia

**Keywords:** *COL3A1*, collagen III, Vascular Ehler-Danlos Syndrome, antisense oligonucleotides

## Abstract

Vascular Ehlers–Danlos syndrome or Ehlers–Danlos syndrome type IV (vEDS) is a connective tissue disorder characterised by skin hyperextensibility, joint hypermobility and fatal vascular rupture caused by *COL3A1* mutations that affect collagen III expression, homo-trimer assembly and secretion. Along with collagens I, II, V and XI, collagen III plays an important role in the extracellular matrix, particularly in the inner organs. To date, only symptomatic treatment for vEDS patients is available. Fibroblasts derived from vEDS patients carrying dominant negative and/or haploinsufficiency mutations in *COL3A1* deposit reduced collagen III in the extracellular matrix. This study explored the potential of an antisense oligonucleotide (ASO)-mediated splice modulating strategy to bypass disease-causing *COL3A1* mutations reported in the in-frame exons 10 and 15. Antisense oligonucleotides designed to redirect *COL3A1* pre-mRNA processing and excise exons 10 or 15 were transfected into dermal fibroblasts derived from vEDS patients and a healthy control subject. Efficient exon 10 or 15 excision from the mature *COL3A1* mRNA was achieved and intracellular collagen III expression was increased after treatment with ASOs; however, collagen III deposition into the extracellular matrix was reduced in patient cells. The region encoded by exon 10 includes a glycosylation site, and exon 15 encodes hydroxyproline and hydroxylysine-containing triplet repeats, predicted to be crucial for collagen III assembly. These results emphasize the importance of post-translational modification for collagen III homo-trimer assembly. In conclusion, while efficient skipping of target *COL3A1* exons was achieved, the induced collagen III isoforms generated showed defects in extracellular matrix formation. While therapeutic ASO-mediated exon skipping is not indicated for the patients in this study, the observations are restricted to exons 10 and 15 and may not be applicable to other collagen III in-frame exons.

## 1. Introduction

Collagen III is considered one of the fibril-forming collagens [1,2,3,4,5] and plays a vital role as a structural collagen fibre in the extracellular matrix (ECM). Collagen III is usually associated with collagen I [6], although present mostly in soft tissues. Collagen III consists of three identical α chains that assemble to form a homo-trimer. Each α chain is encoded by *COL3A1* located on 2q32.2, with the 51 exons spread across 38 kb of genomic sequence being processed into a 5.4 kb mRNA. Collagen III may be divided into three main domains; the C-terminal, alpha helix, and N-terminal domains. The organisation of the C- and N-termini knot-like structures is more complicated and integral to proper folding and fibril formation than the alpha helix region [7]. The collagen triple helix is left-handed and composed of G-X-Y repeats [8], where G indicates glycine and X and Y are other amino acids, usually proline and hydroxyproline. Collagen III is reported to be abundant in embryonic dermis [6] and is broadly detected in many tissues such as skin [9], lung [10], liver [11], uterus [12], vascular tissue [13], bladder [14] and the heart valves [15]. Studies suggest that collagen III might provide ‘flexibility’ in these soft tissues [16], and hence the rupture of internal organs, particularly vascular rupture, is commonly reported in Ehlers–Danlos Syndrome type IV; vascular type (vEDS, EDS type IV; MIM #130050) caused by *COL3A1* mutations.

Vascular Ehler Danlos syndrome is a dominantly inherited disease with an estimated prevalence of 1 in 90,000 [17]. All EDS subtypes are caused by mutations in connective tissue proteins, and joint hypermobility, skin hyperextensibility, and tissue fragility features are common to all subtypes. Congenital hip dislocation and spontaneous pneumothorax are two of the criteria that can distinguish vEDS from other EDS subtypes, based on the International EDS Consortium, 2017. These criteria were developed from the Villefranche diagnostic criteria [18] by including genetic testing via targeted or Sanger sequencing and family history [19]. Following diagnosis of vEDS, genetic counselling and genetic testing of the family members is helpful in disease management and family planning [20]. In particular, pregnant vEDS patients are at the highest risk of distended uterus leading to rupture, with subsequent fatality [21]. Generally, the management of vEDS is limited and no curative treatment is available. vEDS patients are advised to avoid using antiplatelet and/or anticoagulant medications and avoid aggressive physical activity or rapid acceleration/deceleration activities since the vasculature is very fragile. The main medical treatment for vEDS is to manage blood pressure. In addition, vEDS patients are at risk of sudden, unpredictable life-threatening bleeding from vascular rupture. Therefore, apart from blood pressure control, open surgery for vascular ligation is another conservative therapy for vEDS patients [22].

Causative mutations of vEDS can be broadly divided into two groups, according to the proposed pathogenic mechanisms: dominant negative and haploinsufficiency mutations [23,24,25]. The observation that dominant negative mutations result in a shorter life expectancy than haploinsufficiency type mutations [26] suggests that an allele-specific knock-down strategy could potentially delay the age of onset in those with dominant negative mutations. However, the tools and strategies required to efficiently target a single nucleotide difference between healthy and mutant allele transcripts and bring about allele-specific knockdown is possible but remains challenging. Alternatively, removal of selected exons from the transcripts encoded by both alleles, while maintaining the open reading frame and periodicity of the protein (G-X-Y repeats) using antisense oligonucleotide (ASO)-mediated exon skipping, could potentially treat vEDS disease.

ASO-mediated exon-skipping strategies have been successfully developed and are now in clinical use to treat Duchenne muscular dystrophy (DMD) patients. Although induced exon skipping does not provide a cure for DMD, targeted exon skipping reduces disease severity by inducing dystrophin isoforms typically associated with the milder allelic disorder, Becker muscular dystrophy (BMD) [27]. However, supported by extensive clinical datasets on BMD patients, exon skipping is assumed to be most applicable to exons encoding the spectrin-like repeats in the rod domain. Exon skipping cannot be applied to all the *DMD* exons, as those encoding the amino and carboxy terminal domains are essential to function [28]. Although collagen III contains many Gly-X-Y repeat motifs, those exons encoding the termini may not be amenable targets for therapeutic exon skipping, as these encoded domains are essential for normal collagen III folding. However, small in-frame exons encoding the G-X-Y repeat helix region could be potentially amenable targets for exon skipping. One caveat is that, unlike dystrophin, collagen III exists as a homo-trimer, and heterozygous *COL3A1* splice site mutations leading to exon skipping are typically associated with a poor prognosis [29], most likely due to the production of collagen III isoforms of unequal length that affect trimer assembly, exerting a dominant negative effect. We hypothesise that perhaps inducing skipping of the target exon from transcripts encoded by both alleles should produce collagen III monomers of the same length that can be assembled appropriately and retain some function [30].

Our hypothesis is supported by reports of natural in-frame exon skipping from *COL7A1* transcripts that assembled correctly into a homo-trimer and are implicated in milder cases of recessive dystrophic epidermolysis bullosa, a rare form of skin disease [31,32]. Skipping of in-frame exon 73 preserved G-X-Y periodicity and restored the expression of collagen VII [32]. Similarly, an exon-skipping strategy was evaluated in a mouse model of X-linked Alport syndrome, a rare kidney disease caused by mutations in *COL4A5*, and was shown to improve the collagen IV heterotrimer formation and prolong the survival of mice [33]. Despite these studies, there are no reports of exon skipping therapies for Ehlers–Danlos Syndrome type IV.

## 2. Results

### 2.1. Cellular Phenotypes of vEDS Patient Fibroblasts Harbouring COL3A1 Mutations

Two patient fibroblast lines, one with the frameshift mutation c.766delA (located in exon 10) that leads to a premature termination codon (p.Ile256Tyrfs*7) and one carrying a IVS14-2A>G transition that results in exon 15 skipping, were purchased from the Coriell Institute (Camden, NJ, USA).

**Figure 1:**
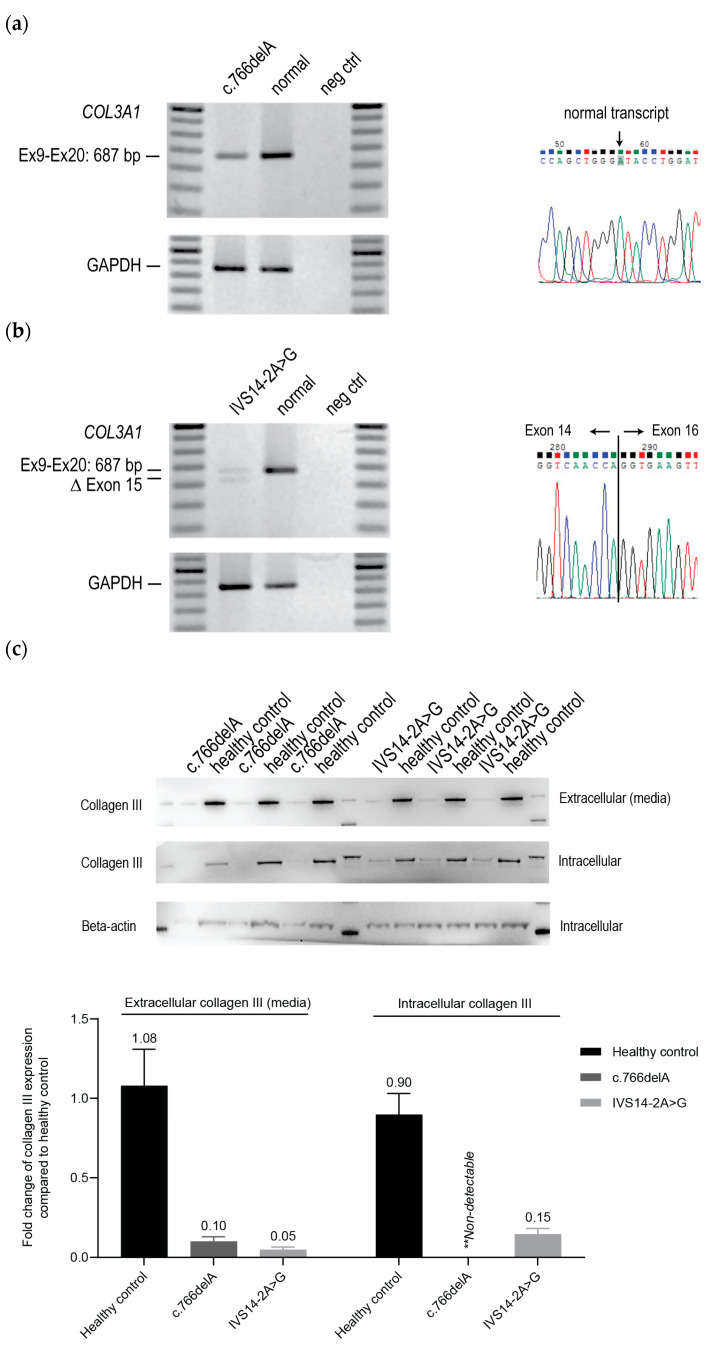

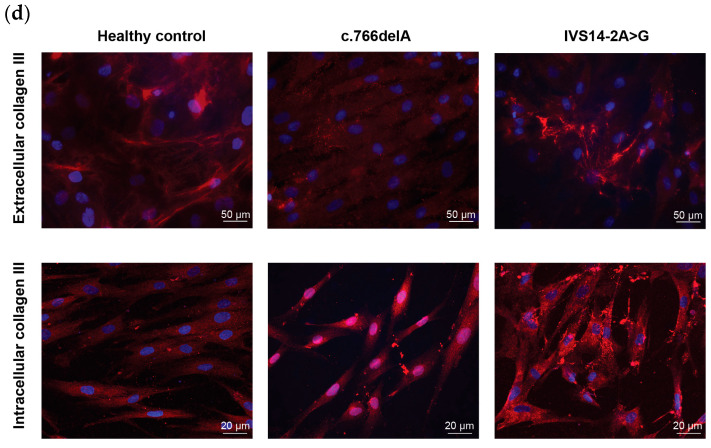
Characterisation of skin fibroblasts derived from two vEDS patients carrying the mutations c.766delA and IVS14-2A>G. (**a**,**b**) RT-PCR amplification and Sanger sequencing of *COL3A1* transcripts (exons 9–20) and *GAPDH* transcript (exon 1–3) from patient fibroblasts. Normal; unaffected healthy control. Neg ctrl; no template control. (**c**) Western blot analysis of collagen III expression in cell lysate (intracellular) and concentrated supernatant (extracellular). Data from triplicate samples are shown. The bar graph represents densitometric analyses (*n* = 3, error bars = SD). (**d**) Immunofluorescence analysis of intracellular and extracellular collagen III in ECM. Red: collagen III. Blue: nuclei.

The expression of *COL3A1* and *GAPDH* as a housekeeping transcript in both patient fibroblast lines was assessed using RT-PCR. The patient cell line carrying the c.766delA mutation showed lower levels of *COL3A1* expression compared to healthy control cells (Figure 1a). Furthermore, only the normal transcript sequence was detected by Sanger sequencing of the amplicon generated by RT-PCR, suggesting very efficient nonsense-mediated decay of the transcript carrying the c.766delA mutation (Figure 1a). This observation suggested that the c.766delA frameshift exerts an effect through a haploinsufficiency mechanism. On the other hand, the patient cells carrying the intronic mutation, IVS14-2A>G, showed in-frame exon 15 skipping (54 bp) upon RNA analysis (Figure 1b); hence, implying a dominant negative disease mechanism. We assessed the intracellular and extracellular content of collagen III produced by both patient-derived cells relative to that from the healthy control donor. Generally, collagen deposition into the ECM only occurs in macromolecular-crowded (MMC) conditions. Hence, we could differentiate intracellular collagen III, the secreted form, and that incorporated into ECM. Both patient-derived cell lines express low levels of intracellular collagen III and secreted lower levels of collagen III than healthy control cells, as determined by western blotting (Figure 1c). Subsequently, intracellular collagen III and ECM deposition were assessed by immunofluorescence on patient and healthy control cell cultures to evaluate the assembly of collagen III. Intracellular collagen III staining in fibroblasts carrying the c.766delA mutation showed a slightly stronger intensity than the healthy control cells. The IVS14-2A>G fibroblasts showed patchy intracellular collagen III staining, suggesting accumulation and/or aggregation, compared to healthy control cells (Figure 1d).

Patient and healthy control fibroblasts were cultured for 5 days under MMC conditions to support extracellular collagen III deposition. Irregular patterns of collagen III deposits were observed in both c.766delA and IVS14-2A>G fibroblast cultures, compared to healthy control fibroblasts (Figure 1d). While the ECM in c.766delA cells appeared as isolated foci, the IVS14-2A>G cells displayed strong but patchy collagen III staining rather than fibrils as observed in the healthy cells. These results suggested that the c.766delA and IVS14-2A>G mutations in *COL3A1* led to not only a reduction in total collagen III but also a defect in the homo-trimer assembly.

### 2.2. ASO-Mediated Exon Skipping to Reframe COL3A1 Transcripts and Assessment of Collagen III Expression in vEDS Patient-Derived Fibroblasts Carrying COL3A1 c.766delA

**Figure 2:**
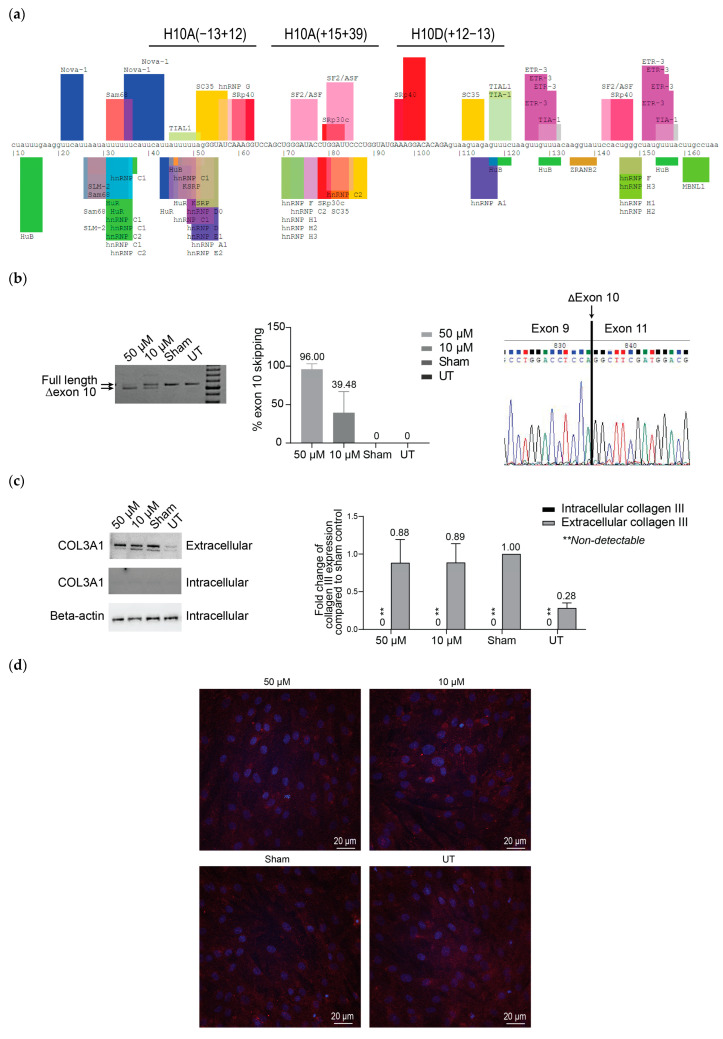
Assessment of *COL3A1* exon 10 skipping and collagen III expression in patient fibroblasts carrying the *COL3A1* c.766delA mutation. (**a**) The exon 10 map indicates the ASO target sites and the exon-splicing enhancer and silencer motifs predicted by Splice Aid. Uppercase letters indicate exonic nucleotides and lowercase letters intronic nucleotides. The image is adapted from the original Splice Aid output (http://www.introni.it/splicing.html, accessed on 19 March 2024). (**b**) RT-PCR analysis of *COL3A1* mRNA across exons 1–14. (**c**) Western blot analysis of intracellular and secreted collagen III protein. Collagen III is detected at top band; ~140 kDa, however, the presence of lower band is likely a non-specific band due to prolonged image exposure. (**d**) Immunofluorescence staining of collagen III deposition, (confocal images) in patient fibroblast cultures, transfected with the exon-skipping PMO cocktail [COL3A1_H10A(+15+39) and COL3A1_H10D(+12−13)] at 50 µM and 10 µM for four days. The bar graphs show average values from three independent transfections (*n* = 3, error bar = SD). Red: collagen III. Blue: nuclei.

Since we found reduced levels of *COL3A1* transcript and intracellular and extracellular collagen III production in the patient fibroblasts carrying the c.766delA mutation, we hypothesized that bypassing the frameshift mutation located in exon 10 by ASO-mediated exon skipping and reframing the *COL3A1* transcript could restore some functional collagen III expression. We designed three ASOs targeting exon 10 (Figure 2a), synthesized as 2′-*O*-methyl modified nucleotides (2′OMe) on a phosphorothioate (PS) backbone and assessed the exon skipping in patient-derived fibroblasts (Supplementary Figure S1). Unfortunately, no single ASO treatment could excise exon 10 (54 bp) and a combination of two ASOs (COL3A1_H10A(+15+39) and COL3A1_H10D(+12−13)) was required to induce exon 10 skipping. Both sequences were subsequently purchased as Phosphorodiamidate morpholino oligomers (PMOs) for further evaluation.

We transfected patient fibroblasts carrying the frameshift mutation c.766delA with the PMO cocktail to induce exon 10 skipping and assessed collagen III production and assembly. Very efficient exon 10 skipping (~96%) was achieved by transfection at a final concentration of 50 µM, and only 39.5% exon 10 skipping was observed at 10 µM (Figure 2b). Since the haploinsufficiency mutation causes a low amount of collagen III production and the majority of collagen III should be secreted, this has led to a barely detectable amount of intracellular collagen III protein (Figure 2c). However, no detectable changes in intracellular collagen III expression or collagen III secretion were observed in *COL3A1* PMO-treated cells when compared to the sham PMO-treated cells. The expression of intracellular collagen III, as determined by western blotting, was normalized to beta-actin expression, but as there is no equivalent housekeeping protein for secreted collagen III, this was normalized to the total protein in the cell lysate [34]. The immunofluorescent detection of collagen III correlated with that demonstrated by western blotting, which showed no difference in collagen expression between untreated cells and cells showing exon 10 skipping (Figure 2d).

### 2.3. ASO-Mediated Exon Skipping to Reframe COL3A1 Transcripts and Assessment of Collagen III Expression in vEDS Patient-Derived Fibroblasts Carrying COL3A1 IVS14-2A>G

**Figure 3:**
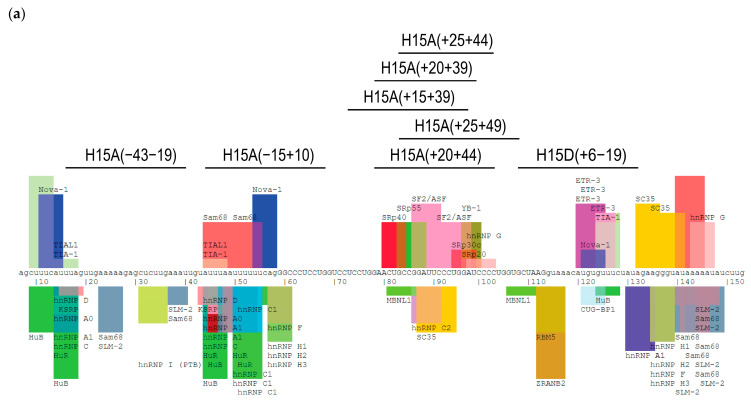

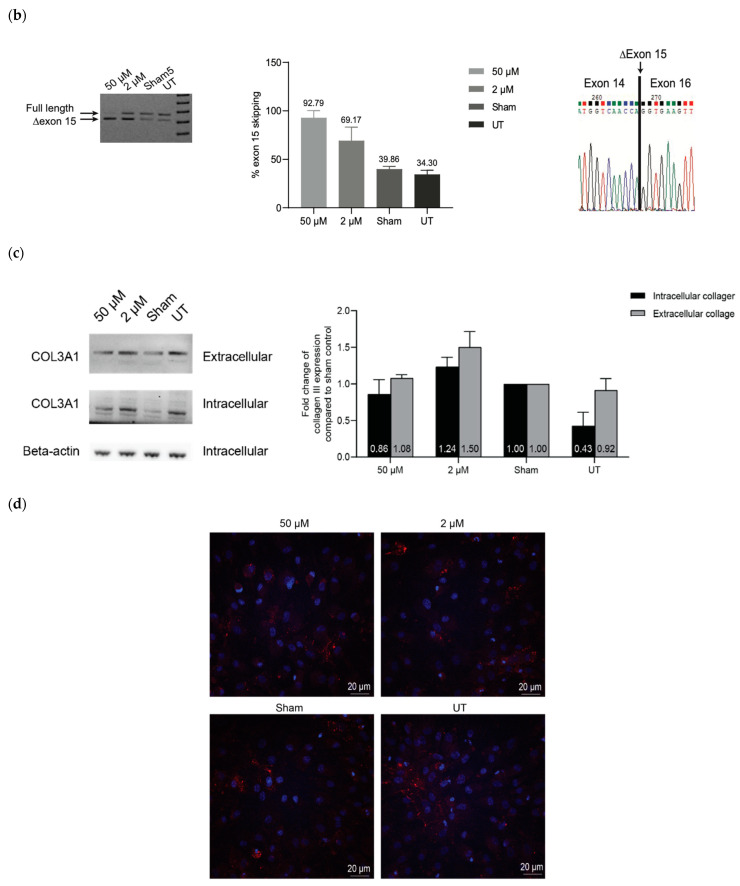
Analysis of *COL3A1* exon 15 skipping, collagen III expression and deposition in IVS14-2A>G patient fibroblasts: (**a**) The exon 15 map indicates the ASO target sites within exon 15 and the exon-splicing enhancer and silencer motifs predicted by Splice Aid output (http://www.introni.it/splicing.html, accessed on 19 March 2024). Uppercase letters indicate exonic nucleotides and lowercase letters intronic nucleotides. The image is adapted from the original Splice Aid output (splice aid). (**b**) RT-PCR analysis of *COL3A1* mRNA across exons 9–20. (**c**) Western blot analysis of intracellular and secreted collagen III protein from patient fibroblast cultures transfected with COL3A1_H14A(+20+44) PMO at 50 µM and 2 µM, at day 4. Collagen III is detected at top band; ~140 kDa, however, the presence of lower band is likely a non-specific band due to prolonged image exposure. The statistical analysis was performed on data from three independent transfections (*n* = 3). (**d**) Immunofluorescence staining of collagen III deposition (confocal images). Red: collagen III. Blue: nuclei.

The IVS14-2A>G mutation results in the loss of *COL3A1* exon 15 (54 bp) from the mature transcript and manifests with reduced expression of intracellular collagen III, irregular assembly and reduced deposition of collagen III in the ECM. The presence of collagen III of unequal lengths, one encoded by the normal healthy allele and the other missing exon 15, may have led to improper assembly of collagen III homofibrils and defective ECM deposition. Hence, we investigated whether inducing exon 15 skipping from the transcript encoded by the normal allele would produce collagen III of identical length that could restore proper assembly. We assessed 8 ASOs (Figure 3a) to induce skipping of exon 15 in patient-derived fibroblasts and identified COL3A1_H15A(+20+44) as the top candidate and purchased the sequence as a PMO (Supplementary Figure S2).

Transfection of the PMO into patient fibroblasts at 50 µM induced efficient skipping of exon 15 (92.8%) from the normal *COL3A1* allele transcript (Figure 3b). However, intracellular collagen III and collagen III secretion remained unchanged (Figure 3c). Immunofluorescent staining also indicated that collagen III deposition in transfected patient cells was unchanged (Figure 3d).

## 3. Discussion

Antisense oligomer-mediated exon-skipping strategies that maintain the open reading frame and periodicity of the protein have been shown to be a viable therapeutic approach for diseases caused by defects in the genes-encoding proteins with repeat motifs such as *DMD* [35], *COL7A* [32] and *COL4A5* [33] (MIM #300377, MIM #226600, MIM #301050, respectively). Likewise, collagen III is a structural protein that is encoded by *COL3A1* which contains mostly in-frame exons, making an ASO-induced exon-skipping strategy promising for vEDS. Therefore, this study explores exon skipping as a plausible therapy for vEDS. The removal of redundant exon(s) from the *COL3A1* transcripts may rescue extracellular collagen III expression, which is a hallmark of a disease phenotype. We hypothesize that removal of one of these in-frame exons from the transcripts encoded by both alleles could preserve protein periodicity and functionality. We chose two representative majority type of mutations including haploinsufficiency and dominant negative to explore which type of mutation can be beneficial for ASO-induced restoration of open reading frame.

Two patient fibroblast lines were studied, one with a haploinsufficiency mutation within exon 10 (c.766delA) and the other with a dominant negative mutation causing exon 15 skipping (IVS14-2A>G). Firstly, we confirmed the mutation that has been reported on the Coriell Institute website (https://www.coriell.org/, accessed on 19 March 2024). A patient fibroblast carrying c.766delA is predicted to cause frameshift mutation leading to nonsense-mediated decay. We observed a restore of *COL3A1* transcript following the treatment of cycloheximide; a nonsense-mediated decay inhibitor (Supplementary Figure S3), hence, further confirmed that *COL3A1* haploinsufficiency is the underlying mechanism leading to vEDS in this patient (c.766delA). On the other hand, an in-frame exon 15 skipping was detected in a patient fibroblast carrying IVS14-2A>G with a reduction in total *COL3A1* transcript compared to a healthy control fibroblast. We have confirmed that this reduction in *COL3A1* transcript is not from nonsense-mediated decay (Supplementary Figure S3). The level of *COL3A1* transcript did not increase after treating with cycloheximide, which clearly inhibited nonsense-mediated decay of *HNRNPD* transcript. Hence, it is likely to be a variation of *COL3A1* expression among individuals which may occur following different conditions such as injury and aging [36,37]. Both patient fibroblasts present with a reduction in collagen III protein compared to healthy control fibroblast as detected by western blot and immunofluorescence assay.

We identified ASO sequences that efficiently induced target exon skipping and assessed collagen III protein expression by western blotting and collagen III assembly by immunofluorescence staining after induced exon skipping. A combination of western blot and immunofluorescence analyses allowed us to assess not only the changes in total intracellular and extracellular collagen III expression but also the collagen III homotrimer/fibril assembly. The analyses minimize any misinterpretation of increased collagen III expression due to collagen III aggregation/accumulation instead of the rescue effect of collagen III following ASO treatment. However, no apparent phenotype rescue was observed in patient cells afterwards, despite achieving around 90% exon skipping with no reduction in RNA expression (Supplementary Figure S4). This result may be explained by the possibility that (i) exon 10 and exon 15 encode amino acids that undergo post-translational modifications crucial for collagen III assembly and/or (ii) skipping of exon 10 or 15 altered amino acids at the splice junctions, leading to interference with flexibility and trimerization, resulting in a disease-like phenotype at the selected time of analysis. Since the observed cell health was compromised starting at day 6, day 4 was selected as the best time point to assess collagen III protein expression in this study (Supplementary Figures S5 and S6).

Collagen III is a fibrillar type of collagen composed of 51 exons, where exon 5 to exon 47 encode for the G-X-Y repetitive region and are all in-frame within this domain [7]. This makes the *COL3A1* transcript theoretically ideal for exon-skipping strategies while maintaining the periodicity of G-X-Y repeats. However, our study revealed that even highly efficient exon skipping could not address mutations that cause vEDS, despite the fact that collagen III forms homotrimers and the induced transcripts should be translated into the same protein isoform. The nature of fibrillar collagen III may mean that the correct protein folding is essential and there is no tolerance for changes. This could be the reason why the phenotype is much milder in patients with null mutations than those with substitutions or splice-site mutations leading to exon skipping [29]. On the other hand, the collagen IV family are non-fibrillar, contain G-X-Y repeats and are a major component of the basement membrane, forming a chicken-wire like meshwork. This structure possibly allows some tolerance to structural changes such as imperfections [38]. Furthermore, a splice site mutation in the *COL4A5* transcript appears to be less severe than mutations causing truncation in the X-linked Alport syndrome [39]. This indicates that even though most collagens share the same structure of G-X-Y repeats, the level of tolerability to changes in the protein structure may vary. This, therefore, affects the therapeutic approach to take, especially when considering a RNA modulating strategy such as exon skipping. These findings further highlight the importance of protein studies in the exon-skipping strategy.

Glycine, or G, is the smallest amino acid and is present in the innermost of every third position, allowing for coiling of the coiled-coil structure. The proline at the ‘X’ position is essential for collagen interchain hydrogen bonding, N-H_(Gly)_···O=C_(X)_, and triple helix stability. Post-translational modifications such as proline hydroxylation, lysine hydroxylation and hydroxylysine glycosylation are crucial for collagen thermal stability [40,41] and fibril cross-linking. Loss of hydroxyproline or mutation in proline-4-hydroxylase enzyme appears to affect the collagen trimerization [42]; however, the loss of glycosylation or mutations that affect this site have never been reported for collagen III. Glycosylated residues are reported to interact with the endocytic mannose receptor family in collagen IV, but the mechanism is still unclear [43]. The amino acids encoded by exon 10 contain two hydroxyproline sites and the only glycosylation site in collagen III (Supplementary Table S1).

In addition, it appears that skipping of some triple-helix-region exons might yield protein isoforms that interfere with the formation of interchain hydrogen bonds in the homo-trimer. Interchain hydrogen bonds are typically formed between the amide group of glycine and the carboxy group of proline; however, Hua et al. found that arginine can also form such bonds [44]. Both exons 10 and 15 encode proline and hydroxyproline, residues that are known to be important for interchain hydrogen-bond formation. Although not all hydroxyproline residues form interchain hydrogen bonds, the remaining non-hydrogen bond forming hydroxyproline residues are important for helix formation in fixing the angle of the C-N peptidyl-proline or peptidyl-hydroxyproline (cis-trans isomerization of N-terminal peptide bond in proline and hydroxyproline) bonds that allow the polypeptide chains to fold into a helical formation [45]. Consequently, the loss of any hydroxyproline sites encoded within an exon targeted for exon skipping might affect trimerization. Nevertheless, additional studies are required to confirm this possibility, such as collagen folding assays [46,47].

During the trimerization process, 3.2 amino acids are involved in one turn of the collagen helix. One residue is always glycine, as it is the smallest amino acid and is required to fit into the innermost space of the helix to accommodate the other two amino acids in the turn. While removal of exons 10 or 15 maintains the reading frame of the encoded G-X-Y repeats, the change of X or Y to Z amino acids might still disrupt the overall helical secondary structure. Skipping of exon 10 changes the alignment of [hydroxyproline-glycine-isoleucine] to [hydroxyproline-glycine-phenylalanine] and skipping of exon 15 changes the alignment of [hydroxyproline-glycine-proline] to [hydroxyproline-glycine-glutamic acid] (Supplementary Figure S7). A single amino acid change in the plane might affect trimerization due to amino acid property changes and the side chain interfering with trimerization and thereby cause a disease-like phenotype.

This study explored ASO-mediated skipping of two in-frame *COL3A1* exons as potential strategies to treat Vascular Ehlers–Danlos syndrome. While efficient skipping of the target exons was achieved, our data do not support skipping of *COL3A1* exons 10 or 15 as a viable treatment. However, each in-frame exon carrying a disease-causing mutation would need to be explored for exon skipping suitability, post-translational modification and protein folding. Despite the exon-skipping strategy maintaining the G-X-Y repeat periodicity, loss of a single exon could still affect the formation of interchain hydrogen bonds. Furthermore, the roles and functions of collagen III post-translational modifications, such as glycosylation and helical hydroxylysine, remain unclear, as does the full scope of collagen III interactions with other molecules. A detailed computational analysis of collagen III folding may provide new insights into these relationships, as would protein interaction studies.

ASOs have been developed to treat what was considered an untreatable disease, DMD, arising from a missing/non-functional dystrophin. However, the delivery of ASOs remains the main issue for non-liver targets and the cost of the drugs is too high to be affordable without government medical benefits or insurance cover. This cost would be even higher if more than one ASO (as observed in exon 10 skipping in this study) is required to be effective. Other strategies evaluated for vEDS as gene replacement or allele silencing were also considered very challenging for vEDS. Perhaps CRISPR/Cas gene editing can be made to be very safe and specific, although one would anticipate great challenges in specificity when targeting the collagen genes.

## 4. Materials and Methods

### 4.1. Antisense Oligonucleotide Design and Synthesis

To induce exon skipping, ASOs were designed to anneal to ESE motifs, as predicted by the online prediction tool, SpliceAid (http://www.introni.it/splicing.html, accessed on 19 March 2024). Oligonucleotide nomenclature is based on that described by Mann et al. and modified by Aung-Htut et al. [48], indicating the intron:exon, exon or exon:intron annealing coordinates in the target pre-mRNA. In this study, the 2′OMe PS-ASOs were purchased from TriLink Biotechnologies, SanDiego, CA, USA and PMOs were purchased from Gene Tools LLC, Philomath, OR, USA. The ASO sequences used in this study are shown in Table 1.

### 4.2. Cell Culture and Transfection

Patient fibroblast carrying *COL3A1* c.766delA mutation and patient fibroblast carrying *COL3A1* IVS14-2A>G were purchased from Coriell Cell repositories (Camden, NJ, USA). Healthy control primary human dermal fibroblasts, prepared from a skin biopsy derived from a healthy volunteer, with informed consent and approved by the Murdoch University human ethics committee (#2013/156; #2017/101), were cultured in Dulbecco’s modified Eagle medium (DMEM), supplemented with L-Glutamine (Gibco; Thermo Fisher Scientific, Scoresby, Australia) and 10% foetal bovine serum (FBS) (Thermo Fisher Scientific, Australia). Phosphorodiamidate morpholino oligomers were introduced into approximately 300,000 cells by Neon electroporation using the Neon electroporation transfection kit system (Thermo Fisher Scientific) using a 1 × 20 ms pulse at 1650 V in a 10 µL tip at the concentrations indicated, following the manufacturer’s instructions. Prior to electroporation, PMO solutions were warmed for 5 min at 37 °C. Transfected cells were incubated in DMEM/F12 media (Gibco) with 2% human serum (Sigma-Aldrich, Castle Hill, NSW, Australia) in 12-well plates for protein analysis and coverslip (13 mm^2^) for ECM staining at 37 °C in 5% CO_2_ for 24 h before harvesting and extraction of RNA. For 2′OMe PS-ASO transfection, fibroblasts were seeded overnight in a 24-well plate to reach 70–80% confluency before transfection with lipofectamine 3000 (Life Technologies, Melbourne, Australia) following the manufacturer’s protocol. In the transfection of 2′OMe PS-ASO cocktails (combinations of more than one 2′OMe PS-ASO), each 2′OMe PS-ASO was incubated with lipofectamine 3000 individually and mixed as a cocktail after 10 min incubation. Individual liposome-2′OMe PS-ASO complexes were combined to 500 µL, added to each well, and incubated for 24 h before harvesting the cells for preparation of RNA.

### 4.3. RNA Harvest and RT-PCR

Total RNA was extracted using the MagMAX-96 Total RNA Isolation Kit (AM1830; Life Technologies) according to the manufacturer’s instructions and included the DNase treatment step. SuperScript III One-Step RT-PCR System (Life Technologies) was used to amplify *COL3A1* transcripts from 50 ng of total RNA in a single step. The PCR conditions and primers used to assess splice modulation across *COL3A1* transcripts are summarized in Table 2.

Amplified RT-PCR products were fractionated on 2% agarose gels in Tris-acetate ethylenediaminetetraacetic acid (EDTA) buffer, stained with RedSafe (iNtRON Biotechnology, Inc., Burlington, MA, USA), and images were captured using a Fusion-FX gel documentation system (Vilber Lourmat, Marne la Vallee, France) using Fusion Capt software (version 17.03). Relative transcript abundance was estimated by densitometry using ImageJ (version 1.8.0_201). The amplicons were isolated by band-stab PCR [49] using AmpliTaq Gold DNA Polymerases (Thermo Fisher Scientific) according to the annealing profile of each primer pair, followed by template preparation using Diffinity Rapidtip for PCR purification (Diffinity Genomics, Inc., West Henrietta, NY, USA). The identities of amplicons were confirmed by Sanger sequencing at the Australian Genome Research Facility Ltd. (Nedlands, Australia).

### 4.4. Protein Harvest and Western Blot

#### 4.4.1. Cell Lysate Preparation

A total of 200,000 fibroblasts were plated into 12-well plates with 600 μL ECM culture medium (culture media containing ascorbic acid) per well. To stimulate post-translational modification (hydroxylation), ECM culture medium with freshly prepared ascorbic acid was added every other day at a final concentration of 50 µg/mL. Spent culture medium was collected in a 1.5 mL tube, and the adherent cells in the plate were washed with phosphate-buffered saline (PBS) on day 4 after transfection. The 1× EDTA-free protease inhibitor (PI) (Sigma-Aldrich) was diluted in PBS (from 25× stock), and 40 µL was added to each well. The cells were scraped and collected into a 1.5 mL tube. The SDS was added to each sample to a final concentration of 5% and the samples were sonicated for six one-second pulses, followed by centrifugation at 14,000× *g* for 3 min. Samples were evaluated for protein content by Pierce BCA protein assay kit (Thermo Fisher Scientific) following the manufacturer’s protocol.

#### 4.4.2. Spent Media Preparation

The medium from each sample well was collected and concentrated in a 10 kDa cut-off protein concentrator column (PALL, Port Washington, NY, USA) by centrifugation at 14,000× *g*, for 20 min, at 4 °C. The flow-through was discarded and all retentate was collected (around 30–50 µL), and SDS was added to each sample to a final concentration of 5%. Samples were evaluated for protein content by Pierce BCA protein assay kit (Thermo Fisher) following the manufacturer’s protocol.

#### 4.4.3. Western Blot

An estimated 25 µg of protein from each sample was loaded on a NuPAGE Novex 4–12% BIS/Tris gel (Life Technologies) and electrophoresed at 200 V for 60 min. An aliquot of Kaleidoscope marker (Biorad) was loaded to visualize separation, and Magic Mark molecular weight marker (Biorad) was used to estimate protein size. The proteins were transferred onto Pall Fluorotrans polyvinylidene fluoride (PVDF) membranes at 350 mA for 90 min in Western transfer buffer (25 mM Tris, 192 mM glycine, 0.01% SDS). Following protein transfer, the membranes were blocked with 5% skimmed milk in 1× TBST for 1 h at room temperature and then incubated with collagen III (1:1000, GeneTex, Irvine, CA, USA) and Beta-actin (1:50,000, Sigma, Castle Hill, NSW, Australia) primary antibodies prepared in 5% skimmed milk in 1× TBST overnight at 4 °C. Membranes were then washed three times in 1× TBST for 15 min each wash, followed by incubation with HRP-labelled secondary antibody prepared in skimmed milk in 1× TBST. The membranes were then washed three times in 1× TBST, each wash for 15 min. The signal was detected using Immobilon Western Chemiluminescent HRP substrate (Merck Millipore, Bayswater, VIC, Australia) following the manufacturer’s protocol. Western blot images were visualized on a Vilber Lourmat Fusion FX system using fusion software (version 17.03), and ImageJ analysis software (version 1.8.0_201) was used for image analysis.

### 4.5. Fibroblast-Derived Extracellular Matrix

Fibroblast-derived extracellular matrix was prepared under macromolecular crowding conditions following the protocol developed by Kumar et al. [50], using Ficoll^®^ as a macromolecular crowding molecule. Small coverslips (13 mm^2^) were placed into 24-well plates and sterilized under UV light for 30 min. Fibroblasts were plated onto coverslips at a density of 15,000 cells/cm^2^ before incubation overnight in DMEM: Hams F12 (3:1), supplemented with 2% human serum and 50 µg/mL ascorbic acid. Media was changed to DMEM:Hams F12 (3:1) with 37.5 mg/mL Ficoll 70 (Sigma) and 25 mg/mL Ficoll 400 (Cytiva Lifesciences, Marlborough, MA, USA), supplemented every other day with 50 µg/mL ascorbic acid to stimulate post-translational hydroxylation and collagen crosslinking. Coverslips were collected on day 6 for immunostaining.

### 4.6. Immunofluorescence Staining

Before immunostaining, coverslips were washed with PBS, fixed in 4% paraformaldehyde for 10 min, and washed again with PBS. For intracellular immunostaining, cells were permeabilized with 0.1% Triton X-100 in PBS for 10 min and washed with PBS. This permeabilization step was not included for ECM staining. Coverslips were blocked with 10% goat serum in PBS for 30 min and incubated with rabbit anti-collagen III (ab7778, Abcam, Melbourne, VIC, Australia) diluted 1:200 in 1% goat serum in PBS overnight at 4 °C. Coverslips were washed with PBS three times for 5 min each wash and then incubated for one hour with Alexa Fluor 488 goat anti-rabbit or Alexa Fluor 568 goat anti-rabbit secondary antibodies (diluted 1:400 in 1% goat serum in PBS) at room temperature. The coverslips were again washed three times with PBS for 5 min, each wash. Hoechst fluorescent dye (1.25%) was used for nuclei staining in the last wash step, before mounting the coverslips on glass slides with ProLong Gold antifade reagent (Thermo Fisher Scientific).

## 5. Conclusions

The results of this study provide further evidence that correct trimerization is crucial to collagen III assembly and secretion. Trimerization of collagen III is proving highly sensitive to perturbation as there is a high incidence of missense mutations reported to cause vEDS. From our observations, both intracellular and extracellular collagen III levels were not improved in vEDS patient-derived cells after ASO-mediated exon skipping, indicating that collagen III modification and assembly within the cells were affected and that these exons are indispensable. However, we cannot conclude that exon skipping is not a viable approach for other *COL3A1* exons.

## Figures and Tables

**Table 1:** ASOs used in this study.

Name	ASO Sequence 5′ --> 3′	Length (bp)	Target *COL3A1* Exon
COL3A1_H10A(−13+12)	ACCUUUGAUACCCUAAAAAUAAUGA	25	10
COL3A1_H10A(+15+39)	ACCAGGGAAUCCAGGUAUCCCAGCU	25	10
COL3A1_H10D(+12−13)	AAACUCUACUUACUCUGUGUCCUUU	25	10
COL3A1_H15A(−43−19)	AUUUCAAGAGCUCUUUUUCAACUAA	25	15
COL3A1_H15A(−15+10)	CAGGAGGGCCCCGAAAAAAUUAAAU	25	15
COL3A1_H15A(+20+44)	GGGGAUCCAGGGAAUCCGGCAGUUC	25	15
COL3A1_H15D(+6−19)	UAUAGAAACACAUGUUUACCUUAGC	25	15
COL3A1_H15A(+25+49)	CACCAGGGGAUCCAGGGAAUCCGGC	25	15
COL3A1_H15A(+15+39)	UCCAGGGAAUCCGGCAGUUCCAGGA	25	15
COL3A1_H15A(+20+39)	UCCAGGGAAUCCGGCAGUUC	20	15
COL3A1_H15A(+25+44)	GGGGAUCCAGGGAAUCCGGC	20	15
Control *	CCTCTTACCTCAGTTACAATTTATA	25	None

* Control ASO targets an intron of the human beta-globin gene. This ASO was purchased from Genetools.

**Table 2:** Primer lists and optimized conditions.

Name	Sequence 5′ --> 3′	Amplicon Size (bp)	Optimized Condition
COL3A1_Ex1F	GCAGGGAACAACTTGATGGTGC	1079	58 °C 30 min
			94 °C 2 min
			94 °C 10 s
COL3A1_Ex14R	GGTTGACCATCACTGCCTCGA		58 °C 10 s
			68 °C 1 min
			for 20 cycles
COL3A1_Ex9F	GACCTGGAGAGCGAGGATTG	687	58 °C 30 min
			94 °C 2 min
			94 °C 10 s
COL3A1_Ex20R	CCTTGCCATCTTCGCCTTTA		58 °C 10 s
			68 °C 1 min
			for 20 cycles
hGAPDH Ex1F	ATGCTGGCGCTGAGTACGT	874	94 °C 2 min
			94 °C 30 s
hGAPDH Ex3R	AGGGGTCTACATGGCAACTG		58 °C 30 s
			68 °C 1 min
			for 20 cycles

## Data Availability

The original contributions presented in the study are included in the article/Supplementary Material, further inquiries can be directed to the corresponding author.

## Supplementary Materials

The following supporting information can be downloaded at: https://www.mdpi.com/article/10.3390/ijms25168816/s1.
