# Supplementary material for: Is Exon Skipping a Viable Therapeutic Approach for Vascular Ehlers–Danlos Syndrome with Mutations in COL3A1 Exon 10 or 15?
Source: Int J Mol Sci. 2024 Aug 13;25(16):8816. doi: 10.3390/ijms25168816 (PMC11354334; doi:10.3390/ijms25168816)
Supplement: Supplementary file 1 [file ijms-25-08816-s001.zip › ijms-3108742-supplementary.pdf]

Supplementary data

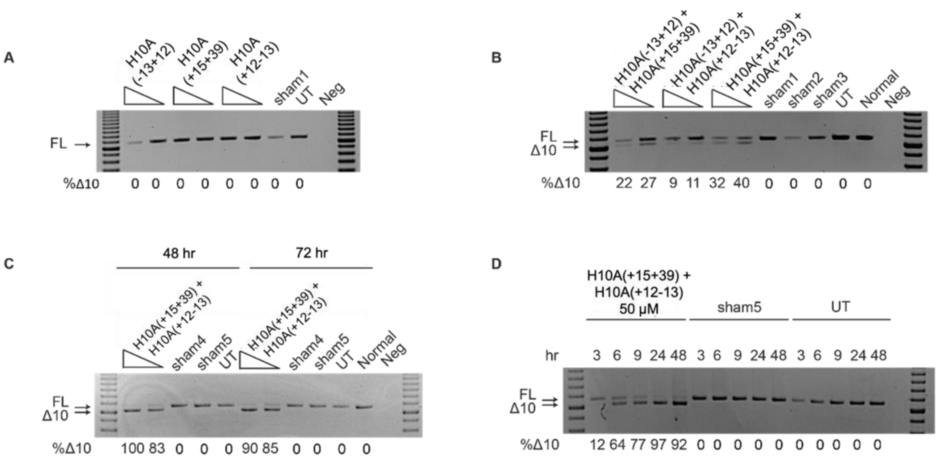

Supplementary Figure S1. ***COL3A1* exon 10 skipping to address the c.766delA mutation.** RT-PCR analysis of *COL3A1* mRNA (exons 1-14) from patient fibroblasts carrying the *COL3A1* c.766delA change, transfected with **(A)** individual 2´OMe PS-AO (200 and 50 nM) for 24 hr, **(B)** 2´OMe PS-AO cocktails (200 and 50 nM) for 24 hr, **(C)** AO6 and AO7 PMO cocktail (100 µM and 20 µM) and **(D)** AO6 and AO7 PMO cocktail (100 µM) for various times.

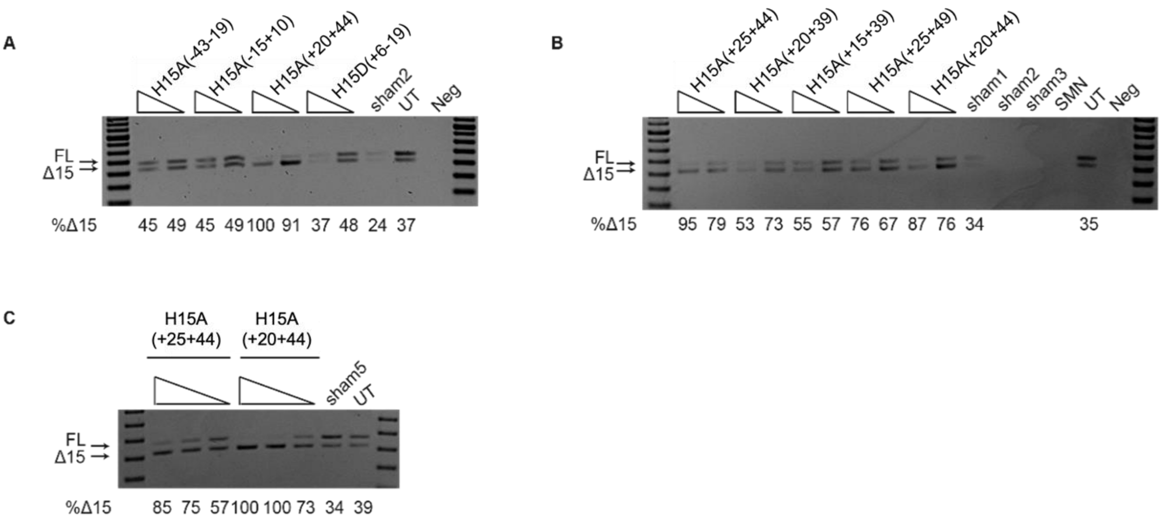

Supplementary Figure S2. ***COL3A1* exon 15 skipping to address the IVS14-2A>G mutation.** RT-PCR analysis of *COL3A1* (exons 9-20) in patient fibroblasts carrying *COL3A1* IVS14-2A>G transfected with (A) 2´OMe PS-ASOs targeted across exon 15 (200 nM and 50 nM) for 24 hr (B) microwalk ASO around COL3A1_H15A(+20+44) (200 nM and 50 nM) for 24 hr (C) PMO COL3A1_H15A(+25+44) and COL3A1_H15A(+20+44) (50, 10, 2 µM) for 24 hr. Sham 1-3 are scrambled sequences synthesized as 2′OMe PS-AOs and Sham 5 is a scrambled sequence synthesized as PMO.

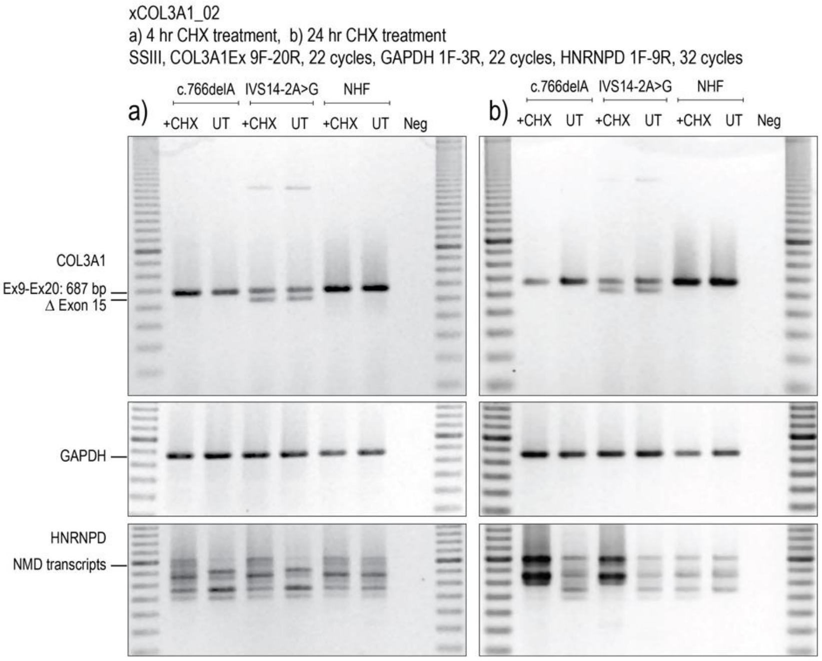

Supplementary Figure S3. Confirmation of nonsense mediated decay transcript by cycloheximide treatment in patient carrying *COL3A1* c.766delA and IVS14-2A>G. RT-PCR analysis of *COL3A1* (exon 9-20) and house-keeping gene *GAPDH* (exon 1-3) following 4-hr (A) and 24-hr (B) of cycloheximide treatment. The transcript *HNRNPD* was used as a control for NMD transcript restoration following a successful cycloheximide treatment.

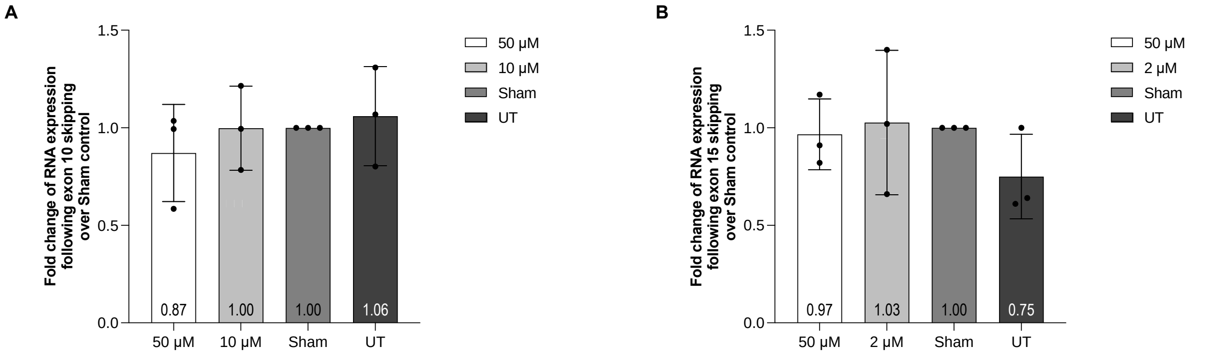

Supplementary Figure S4 Semi-quantitative analysis of RNA expression following exon 10 (A) and 15 skipping (B). RNA expression was semi-quantified following exon skipping by densitometry.

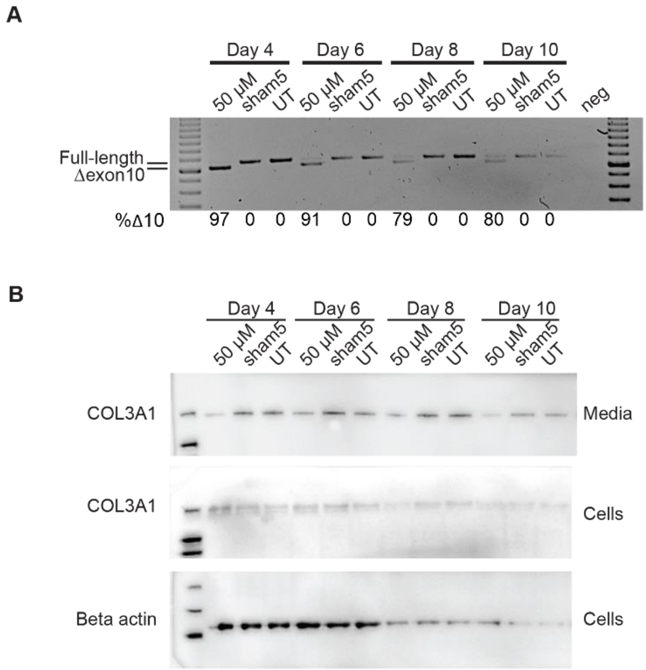

Supplementary Figure S5. Time course optimization for skipping of exon 10 and collagen III detection in c.766delA patient fibroblasts at days 4, 6, 8 and 10 after transfection with PMO COL3A1_H10A(+15+39) and COL3A1_H10D(+12-13). (A) Exon 10 skipping was detected by RT-PCR across *COL3A1* mRNA exons 1-14 in patient fibroblasts. (B) Collagen III expression was determined by western blot analysis after induction of exon 10 skipping in patient fibroblasts. The media was collected to assess extruded collagen III followed by washing with PBS and harvesting of cells to assess intracellular collagen III.

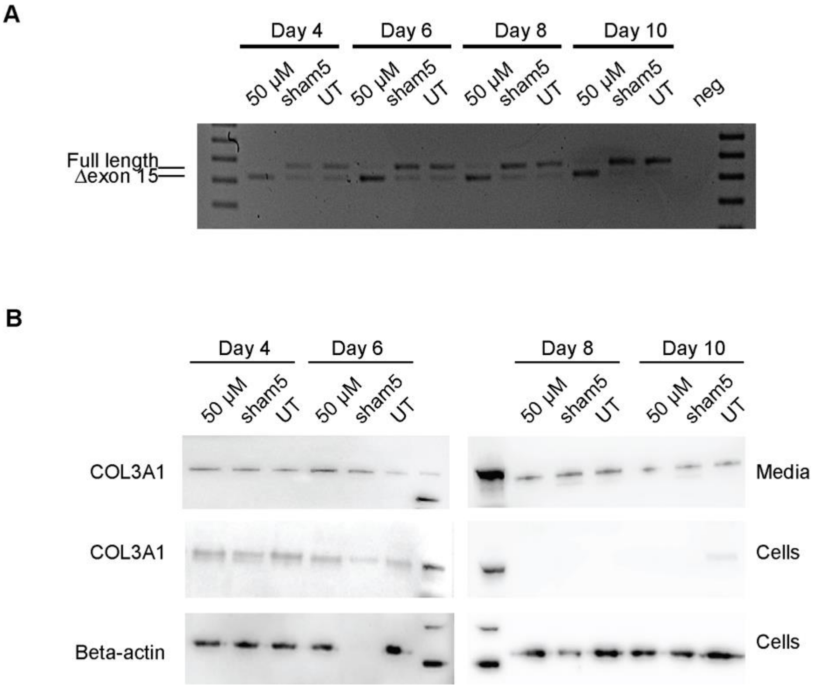

Supplementary Figure S6. Time course optimization for exon 15 skipping and collagen III detection in IVS14-2A>G patient fibroblasts at days 4, 6, 8 and 10 after transfection with PMO COL3A1_H15A(+20+44). (A) Exon 15 skipping was detected by RT-PCR across *COL3A1* mRNA exons 9-20 in patient fibroblasts. (B) Collagen III expression was determined by western blot analysis after induction of exon 15 skipping in patient fibroblasts. The media was collected to assess extruded collagen III followed by washing with PBS and cells scrapping to assess intracellular levels of collagen III.

Supplementary Table S1. Collagen III amino acid and post-translational modifications encoded by the exons targeted for exclusion. The hydroxylation sites are indicated in red text and the glycosylation site is underlined.

Exon	Amino Acids	Posttranslational modifications
10	GIKGPAGIPGFPGMKGHR	Hydroxyproline, glycosylation
15	GPPGPPGTAGFPGSPGAK	Hydroxyproline

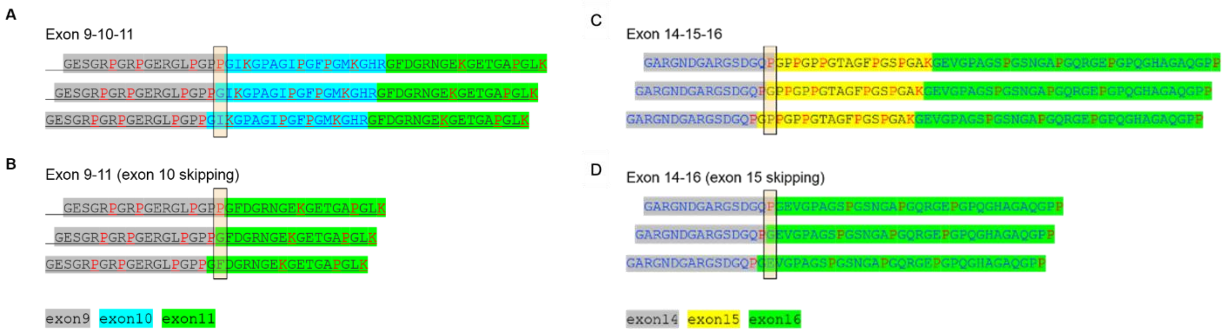

Supplementary Figure S7. The predicted effect of *COL3A1* exon 10 skipping (A and B) and exon 15 skipping (C and D) on homo-trimer alignment. Amino acid alignments for the protein region encoded by exons 9, 10 and 11 (A) and exon 14, 15 and 16 (C). Amino acid alignment of the protein region encoded by the splicing of exon 9 and 11 (B) and 14 and 16 (D) after exon 10 or 15 skipping respectively. Exon 9, 10 and 11 sequences are highlighted in grey, blue and green, respectively. Exon 14, 15 and 16 sequences are highlighted in grey, yellow and green, respectively. The box highlights the alignment of the amino acid in the same turn. Red text indicates hydroxylation sites.
